# Healthcare-associated pneumonia in acute care hospitals in European Union/European Economic Area countries: an analysis of data from a point prevalence survey, 2011 to 2012

**DOI:** 10.2807/1560-7917.ES.2018.23.32.1700843

**Published:** 2018-08-09

**Authors:** Jan Walter, Sebastian Haller, Chantal Quinten, Tommi Kärki, Benedikt Zacher, Tim Eckmanns, Muna Abu Sin, Diamantis Plachouras, Pete Kinross, Carl Suetens, ECDC PPS study group

**Affiliations:** 1 Unit of Healthcare-associated Infections, Surveillance of Antibiotic Resistance and Consumption, Department of Infectious Disease Epidemiology, Robert Koch Institute, Berlin, Germany; 2 Surveillance and Response Support Unit, European Centre for Disease Prevention and Control (ECDC), Stockholm, Sweden; 3 The members of the ECDC PPS study group are listed at the end of the article

**Keywords:** healthcare-associated infections, pneumonia, epidemiology, surveillance, antimicrobial resistance, antibiotic use, infection prevention and control, point prevalence survey, Europe

## Abstract

An aim of the ECDC point prevalence survey (PPS) in European Union/European Economic Area acute care hospitals was to acquire standardised healthcare-associated infections (HAI) data. We analysed one of the most common HAIs in the ECDC PPS, healthcare-associated pneumonia (HAP). Standardised HAI case definitions were provided and countries were advised to recruit nationally representative subsets of hospitals. We calculated 95% confidence intervals (CIs) around prevalence estimates and adjusted for clustering at hospital level. Of 231,459 patients in the survey, 2,902 (1.3%; 95% CI: 1.2–1.3) fulfilled the case definition for a HAP. HAPs were most frequent in intensive care units (8.1%; 95% CI: 7.4–8.9) and among patients intubated on the day of the survey (15%; 95% CI: 14–17; n = 737 with HAP). The most frequently reported microorganism was *Pseudomonas aeruginosa* (17% of 1,403 isolates), followed by *Staphylococcus aure*us (12%) and *Klebsiella* spp. (12%). Antimicrobial resistance was common among isolated microorganisms. The most frequently prescribed antimicrobial group was penicillins, including combinations with beta-lactamase inhibitors. HAPs occur regularly among intubated and non-intubated patients, with marked differences between medical specialities. HAPs remain a priority for preventive interventions, including surveillance. Our data provide a reference for future prevalence of HAPs at various settings.

## Introduction

Healthcare-associated pneumonia (HAP) causes a considerable burden of disease in the European Union/European Economic Area (EU/EEA) [1], which is at least partially preventable [2]. Surveillance of healthcare-associated infections (HAIs) can contribute to prevention by increasing awareness and by providing data for the prioritisation of interventions and for their subsequent evaluation [3].

Point prevalence surveys (PPSs) are a surveillance methodology well-suited to HAI surveillance. They are easier to conduct and much less costly than incidence surveillance of HAIs, even though they have drawbacks in terms of assessing causality and the overrepresentation of patients with long hospital stays [4]. Several PPSs that assessed HAP prevalence have been conducted in Europe and North America [5-17]; however, their methods differed. For example, some PPSs did not specify the number of intubated patients, a group known to be at increased risk for HAP, while others reported cases of lower respiratory tract infections rather than pneumonia, limiting comparability across PPSs.

The European Centre for Disease Prevention and Control (ECDC) PPS of HAIs and antimicrobial use in EU/EEA acute care hospitals from 2011–12 applied a standardised methodology for the surveillance of HAIs, including HAP, throughout the EU, as well as in Croatia (EU Member State since 1 July 2013), Iceland and Norway, referred to herein as the EU/EEA for brevity. This very large study included data on HAIs, as well as on microbiological results and antimicrobial use [18]. Previous publications include a report on the overall analyses of the ECDC PPS [18] and a report on the analysis of data from paediatric patients [19], though HAP were not analysed in detail in these reports. We present the results of an analysis focusing on HAP in EU/EEA acute care hospitals that participated in the ECDC PPS with the aim of providing a reference for the prevalence of HAPs at various settings, which can aid the interpretation of locally collected surveillance data and guide preventive interventions.

## Methods

### Study design

The ECDC PPS aimed to measure the prevalence of HAIs in acute care hospitals with a similar precision in each EU/EEA country. ECDC recommended that countries select hospitals to participate by clustered random sampling, if possible. All acute care hospitals were eligible. Prevalence data on HAIs, antimicrobial use and selected risk factors were collected by trained data collectors on one single day per ward during the 2011–12 survey period (generally outside the winter period of December to March and the summer holiday period of July to August, with peaks of data collection from September to November 2011 and May to June 2012) [18]. Hospitals chose to collect data either at patient level (‘standard’ option) or in partially aggregated form (‘light’ option). Prior to the ECDC PPS, case definitions were developed through international consultations and were tested in a pilot study [20].

To assess the severity of each patient’s underlying disease (standard option only), study staff allocated a McCabe score, classified as either non-fatal, ultimately fatal (expected survival between 1 and 5 years) or rapidly fatal (expected survival less than 1 year) [21].

### Case definition

The ECDC PPS case definition for HAP arises from the ECDC PPS definition of a pneumonia and a HAI [22]:

A pneumonia (Box) was defined as:

‘Active’ on the day of the survey when: signs and symptoms were present on the date of the survey; OR signs and symptoms were no longer present but the patient was still receiving treatment for an infection on the date of the survey;

‘healthcare-associated’ (i.e. HAP) when: the onset of the signs and symptoms was on Day 3 of the current admission or later (with Day 1 being the day of admission); OR the signs and symptoms were present on admission or became apparent before Day 3, and the patient had been discharged from an acute care hospital less than two days before admission.

HAP was stratified into five disease codes, from PN1 to PN5, corresponding to different degrees of microbiological evidence for non-neonatal pneumonia and into ‘Neo-Pneu’ for neonatal pneumonia according to the case definitions shown in the Box.

Box **Case definitions for pneumonia, neonatal pneumonia and intubation-associated pneumonia, ECDC point prevalence survey in acute care hospitals, European Union/European Economic Area^a^, 2011–2012**

**Pneumonia**

Two or more serial chest X-rays or CT-scans with a suggestive image of pneumonia for patients with underlying cardiac or pulmonary disease. In patients without underlying cardiac or pulmonary disease, one definitive chest X-ray or CT-scan is sufficient

AND at least one of the following symptoms

• fever > 38 °C with no other cause;

• leukopenia (< 4,000 WBC/mm^3^) or leucocytosis (≥ 12,000 WBC/mm^3^);

AND at least one of the following (or at least two if clinical pneumonia only = PN4 and PN5):

• new onset of purulent sputum, or change in character of sputum (colour, odour, quantity, consistency);

• cough or dyspnea or tachypnea;

• suggestive auscultation (rales or bronchial breath sounds), ronchi, wheezing;

• worsening gas exchange (e.g. O_2_ desaturation or increased oxygen requirements or increased ventilation demand).

**Microbiological characterisation according to the used diagnostic method**

(i) Bacteriological diagnostic test performed by:

• Positive quantitative culture from minimally contaminated LRT specimen (**PN1**):

 − BAL with a threshold of > 10^4^ CFU/mL or ≥ 5% of BAL obtained cells contain intracellular bacteria on direct microscopic exam (classified on the diagnostic category BAL);

 − protected brush (PB Wimberley) with a threshold of > 10^3^ CFU/mL;

 − distal protected aspirate (DPA) with a threshold of > 10^3^ CFU/mL.

• Positive quantitative culture from possibly contaminated LRT specimen (**PN2**):

 − Quantitative culture of LRT specimen (e.g. endotracheal aspirate) with a threshold of 10^6^ CFU/mL.

(ii) Alternative microbiology methods (**PN3**):

• positive blood culture not related to another source of infection;

• positive growth in culture of pleural fluid;

• pleural or pulmonary abscess with positive needle aspiration;

• histologic pulmonary exam shows evidence of pneumonia;

• positive exams for pneumonia with virus or particular germs (*Legionella* spp., *Aspergillus* spp., mycobacteria, mycoplasma, *Pneumocystis carinii*):

 − positive detection of viral antigen or antibody from respiratory secretions (by e.g. enzyme immunoassay , fluorescent-antibody-to-membrane-antigen, shell vial assay, PCR tests);

 − positive direct exam or positive culture from bronchial secretions or tissue;

 − seroconversion (e.g. for influenza viruses, *Legionella* spp., *Chlamydia* spp.);

 − detection of antigens in urine (*Legionell*a spp.).

(iii) Others:

• positive sputum culture or non-quantitative LRT specimen culture (**PN4**);

• no positive microbiology (**PN5**).

**Neonatal pneumonia**

Respiratory compromise;

AND

New infiltrate, consolidation or pleural effusion on chest X-ray;

AND at least four of:

• temperature > 38 °C or < 36.5 °C or temperature instability;

• tachycardia or bradycardia;

• tachypnoea or apnoea;

• dyspnoea;

• increased respiratory secretions;

• new onset of purulent sputum;

• isolation of a pathogen from respiratory secretions;

• C-reactive protein > 2.0 mg/dL;

• I/T ratio > 0.2.

**Intubation-associated pneumonia**

Pneumonia is defined as intubation-associated if an invasive respiratory device was present (even intermittently) within the 48 hours preceding the onset of infection.

BAL: broncho-alveolar lavage; CFU: colony-forming units; CT: computed tomography; ECDC: European Centre for Disease Prevention and Control; I/T: immature to total neutrophil ratio; LRT: lower respiratory tract; PN: pneumonia; WBC: white blood cells.
^a^European Union countries, plus Croatia (EU Member State since 1 July 2013), Iceland and Norway.

All analyses used the ECDC PPS definition for HAP, which differs from the definitions used in other publications that differentiate hospital- and healthcare-associated pneumonia [22,23].

### Statistical analyses

Prevalence was calculated by dividing the number of patients with HAP by all patients in the hospital at the time of the survey. We included data from both the ‘light’ and the ‘standard’ option for the calculation of the overall and country-specific prevalence estimates. We only included hospitals using the ‘standard’ option for the risk factor analysis. We adjusted the calculation of confidence intervals (CIs) around the HAP prevalence to account for the clustered study design (i.e. the design effect) using the Stata survey command (svy) and specifying the option ‘singleunit (scaled)’ in case there were strata with only one cluster. Pearson chi-squared tests were also adjusted for the design effect using the survey command, and were used to assess associations of covariates with the HAP prevalence. Odds ratios (ORs) were calculated by mixed logistic regression modelling, including a random offset at hospital level to adjust for clustering.

Categories for age, hospital size and medical speciality were chosen to match the main ECDC report [18]. The time between the most recent admission to the surveyed hospital and the onset of symptoms was divided into three categories (≤ 4 days, 4–7 days, ≥ 8 days), partially as done previously [24] and partially to reflect the relatively late onset of disease seen in this dataset. We used Anatomic Therapeutic Chemical classification system (ATC) 3 and 4 categories (https://www.whocc.no/atc_ddd_index) for the analysis of antibiotic use.

All analyses were conducted in Stata 14.1 (College Station, Texas, United States (US)).

Ethical approval was at the discretion of each national public health and government body. Anonymised patient- and institution-level data was shared with ECDC and collaborating partners for this analysis.

## Results

### Healthcare-associated pneumonia prevalence

A total of 231,459 patients from 947 hospitals in 30 EU/EEA countries were included in the dataset. HAP was present in 2,902 patients resulting in a prevalence of 1.3% (95% CI: 1.2–1.3%) among hospitalised patients in acute care hospitals in Europe. The HAP prevalence varied between 0.6% (95% CI: 0.2–1.4%) in Latvia and 3.7% (95% CI: 1.0–12.3%) in Iceland (Pearson chi-squared adjusted for clustering: p value < 0.0001 over all countries) (Figure).

**Figure:**
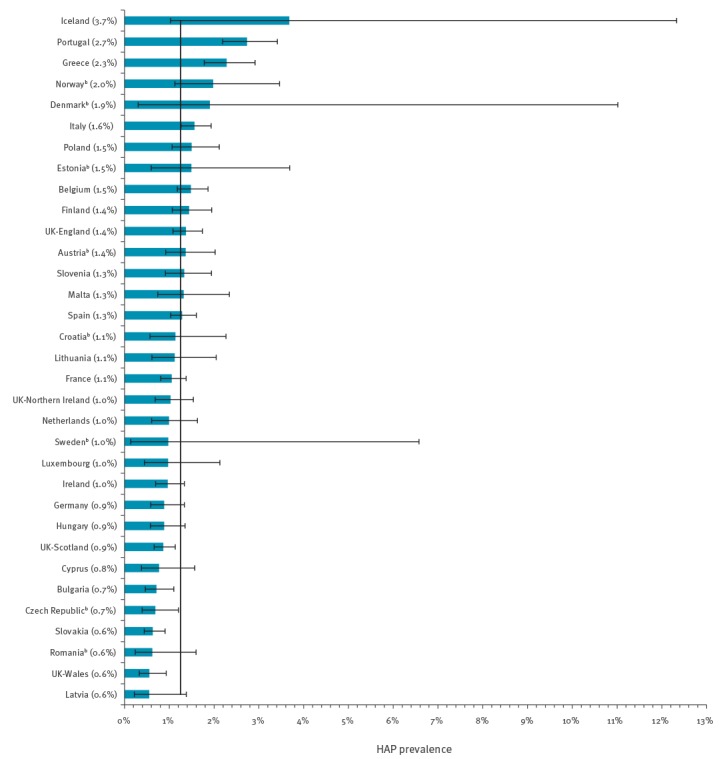
Prevalence of healthcare-associated pneumonia, ECDC point prevalence survey in acute care hospitals, European Union/European Economic Area^a^, 2011–2012 (n = 231,459 patients)

ECDC: European Centre for Disease Prevention and Control; HAP: healthcare-associated pneumonia; UK: United Kingdom.

^a^European Union countries, plus Croatia (EU Member State since 1 July 2013), Iceland and Norway.

^b^Countries with poor or very poor representation of their national sample of participating hospitals, as defined in the ECDC report [18].

Countries are ranked from highest to lowest observed prevalence of HAP, according to the ECDC point prevalence survey case definition, with 95% confidence intervals. The value of each national prevalence is shown in parenthesis adjacent to each country name. The vertical line indicates the overall prevalence of HAP, i.e. 1.25%.

For 2,838 (98%) of the HAP cases, information on whether intubation had been present before the onset of HAP was available. Of these, 947 (33%) had an intubation-associated pneumonia (IAP), while the remaining 1,891 (67%) cases had not been intubated before the onset of their HAP.

The majority (93%, n = 880) of hospitals used the ‘standard’ patient-based surveillance option. They included 215,537 patients, for whom an in-depth analysis is possible. Among these, we found HAP to be significantly associated with older age, male sex, higher McCabe score, intubation and prolonged hospital stay. HAP was most frequent among intensive care unit (ICU) patients, in tertiary care hospitals and in large hospitals (Table 1).

**Table 1:** Characteristics of healthcare-associated pneumonia cases and non-cases collected in the ‘standard’ surveillance option, ECDC point prevalence survey in acute care hospitals, European Union/European Economic Area^a^ 2011–2012 (n = 215,537)

Characteristics	HAP^b^ N	Total^b^ N	HAP prevalence		Odds ratio (95% CI)
			% of all patients (95% CI)	p value	
**Total**	**2,748**	**215,537**			
**Age**					
< 1 month	61	7,592	0.8 (0.5–1.2)	< 0.0001	1.5 (1.1–2.0)
1–11 months	67	5,135	1.3 (1.0–1.8)		2.1 (1.6–2.8)
1–44 years	276	46,838	0.6 (0.5–0.7)		Reference
45–74 years	1,188	88,726	1.3 (1.2–1.5)		2.4 (2.1–2.7)
75–84 years	776	43,665	1.8 (1.6–2.0)		3.2 (2.8–3.7)
≥ 85 years	377	23,319	1.6 (1.4–1.8)		3.1 (2.6–3.6)
**Sex**					
Female	1,024	113,517	0.9 (0.8–1.0)	< 0.0001	0.6 (0.5–0.8)
Male	1,717	101,137	1.7 (1.6–1.8)		Reference
**McCabe score**					
Nonfatal	991	142,925	0.7 (0.6–0.8)	< 0.0001	Reference
Ultimately fatal	855	34,780	2.5 (2.2–2.7)		3.6 (3.3–4.0)
Rapidly fatal	485	11,275	4.3 (3.9–4.8)		6.7 (6.0–7.6)
**Intubated on survey date**					
Yes	737^c^	4,906	15 (14–17)	< 0.0001	18 (17–20)
No	1,999^c^	20,9774	1.0 (0.9–1.0)		Reference
**Length of stay until HAP or until survey dates (days)^d^**					
≤ 3	399	71,000	0.6 (0.5–0.6)	< 0.0001	Reference
4–7	560	57,327	1.0 (0.9–1.1)		1.7 (1.5–1.9)
8–14	494	41,740	1.2 (1.1–1.3)		2.0 (1.8–2.3)
≥ 15	772	43,911	1.8 (1.6–1.9)		3.1 (2.7–3.5)
**Hospital type**					
Primary care	291	31,401	0.9 (0.8–1.1)	< 0.0001	Reference
Secondary care	845	75,275	1.1 (1.0–1.3)		1.3 (1.0–1.6)
Tertiary care	1,383	85,363	1.6 (1.5–1.8)		1.8 (1.5–2.3)
Specialised	121	12,573	1.0 (0.7–1.4)		0.8 (0.6–1.2)
**Hospital size (number of beds)**					
< 200	202	21,200	1.0 (0.8–1.2)	< 0.0001	Reference
200–399	561	50,069	1.1 (1.0–1.3)		1.2 (0.9–1.4)
400–649	644	55,746	1.2 (1.0–1.3)		1.2 (1.0–1.5)
≥ 650	1,341	88,522	1.5 (1.3–1.7)		1.6 (1.3–2.1)
**Speciality**					
Geriatrics	101	8,982	1.1 (0.9–1.5)	< 0.0001	1.0 (0.8–1.2)
Gynaecology/Obstetrics	12	16,493	0.07 (0.04–0.13)		0.1 (0.0–0.1)
Intensive care unit	853	10,504	8.1 (7.4–8.9)		7.1 (6.5–7.8)
Medical	1,138	88,745	1.3 (1.1–1.4)		Reference
Mixed	3	782	0.4 (0.1–1.2)		0.3 (0.1–1.1)
Other	13	1,160	1.1 (0.6–2.2)		1.0 (0.6–1.7)
Paediatrics	60	12,037	0.5 (0.3–0.7)		0.4 (0.3–0.5)
Psychiatrics	9	8,226	0.1 (0.1–0.2)		0.1 (0.0–0.2)
Rehabilitation	28	3,068	0.9 (0.6–1.5)		0.7 (0.5–1.0)
Surgery	530	65,370	0.8 (0.7–0.9)		0.6 (0.5–0.7)

CI: confidence interval; ECDC: European Centre for Disease Prevention and Control; HAP: healthcare associated pneumonia; IQR: interquartile range.

^a^European Union countries, plus Croatia (EU Member State since 1 July 2013), Iceland and Norway.

^b^Columns may not tally to the total due to missing data.

^c^Eighty percent (587/737) of patients with intubation on the survey date and 15% (303/1,999) of patients without incubation on the survey date were classified as intubation-associated pneumonia.

^d^Excluding patients with HAP at admission.

### Laboratory data

Five hundred and sixteen (18%) HAP cases were defined as laboratory-confirmed based on the case definition code (PN1–PN3); 2,258 (78%) cases were non-laboratory confirmed (PN4 or PN5) and 64 (2%) were classified as neonatal pneumonia. The classification was missing for 64 (2%) HAP cases. The proportion of laboratory-confirmed cases (PN1–PN3) varied between 55% in Norway and 1% in Cyprus; it was higher in ICU patients than in non-ICU patients (32% vs 12%, p < 0.001).

One thousand one hundred and sixty-six (40%) patients with HAP had one or more microbiological result reported to ECDC (including all case definition codes except PN5). Of these, 103 (9%) patients had sterile samples. Of the remaining 1,063 patients, 478 (45%) were classified as PN1‒PN3, 558 (52%) as PN4, 16 (2%) as neonatal pneumonia and 11 (1%) as unspecified. From the 1,166 patients, 1,403 isolates of microorganisms were identified. The isolated microorganisms were mostly bacteria (90%; 1,257 isolates), and were rarely fungi or parasites (10%; 143 isolates) or viruses (< 1%; 3 isolates). The most frequently reported microorganism was *Pseudomonas aeruginosa* (17%), followed by *Staphylococcus aure*us (12%) and *Klebsiella* spp. (12%) (Table 2).

**Table 2:** Microorganisms isolated in healthcare-associated pneumonia cases according to association with intubation, time of onset and diagnostic category, ECDC point prevalence survey in acute care hospitals, European Union/European Economic Area^a^, 2011–2012 (n = 1,403 isolates)

Microorganism	All HAP cases		Intubation-associated				Time of onset^b^						Diagnostic category			
			Yes		No/Unknown		≤ 4 days		5–7 days		≥ 8 days		PN1–PN3^c^		PN4^c^	
	n	%	n	%	n	%	n	%	n	%	n	%	n	%	n	%
**Total**	**1,403**	**100**	**827**	**100**	**576**	**100**	**199**	**100**	**184**	**100**	**718**	**100**	**648**	**100**	**725**	**100**
**Gram-positive cocci**	**265**	**19**	**126**	**15**	**139**	**24**	**53**	**27**	**42**	**23**	**115**	**16**	**124**	**19**	**132**	**18**
*Staphylococcus aureus*	168	12	79	10	89	15	32	16	24	13	76	11	77	12	86	12
Coagulase-negative staphylococci	22	2	9	1	13	2	3	2	3	2	11	2	12	2	8	1
*Streptococcus* spp.	35	2	13	2	22	4	14	7	9	5	9	1	12	2	22	3
*Enterococcus* spp.	31	2	18	2	13	2	1	1	5	3	17	2	19	3	12	2
Other Gram-positive cocci	9	1	7	1	2	0	3	2	1	1	2	0	4	1	4	1
**Gram-negative cocci**	**18**	**1**	**5**	**1**	**13**	**2**	**4**	**2**	**3**	**2**	**10**	**1**	**6**	**1**	**12**	**2**
**Gram-positive bacilli**	**6**	**0**	**4**	**0**	**2**	**0**	**0**	**0**	**1**	**1**	**2**	**0**	**4**	**1**	**2**	**0**
**Enterobacteriaceae**	**454**	**32**	**275**	**33**	**179**	**31**	**69**	**35**	**59**	**32**	**225**	**31**	**186**	**29**	**254**	**35**
*Citrobacter* spp.	12	1	8	1	4	1	1	1	2	1	3	0	7	1	5	1
*Enterobacter* spp.	71	5	43	5	28	5	13	7	8	4	39	5	20	3	47	6
*Escherichia coli*	120	9	68	8	52	9	16	8	19	10	58	8	45	7	72	10
*Klebsiella* spp.	164	12	102	12	62	11	28	14	15	8	81	11	68	10	91	13
*Proteus* spp.	33	2	22	3	11	2	4	2	4	2	15	2	21	3	11	2
*Serratia* spp.	37	3	24	3	13	2	4	2	10	5	18	3	15	2	21	3
Other Enterobacteriaceae	17	1	8	1	9	2	3	2	1	1	11	2	10	2	7	1
**Non-fermenting Gram-negative bacteria**	**4,444**	**32**	**302**	**37**	**142**	**25**	**39**	**20**	**51**	**28**	**264**	**37**	**222**	**34**	**217**	**30**
*Acinetobacter* spp.	136	10	106	13	30	5	7	4	21	11	81	11	63	10	72	10
*Pseudomonas aeruginosa*	244	17	154	19	90	16	29	15	22	12	141	20	129	20	112	15
*Stenotrophomonas maltophilia*	47	3	30	4	17	3	3	2	4	2	29	4	19	3	27	4
Other *Pseudomonadaceae*	17	1	12	1	5	1	0	0	4	2	13	2	11	2	6	1
**Other Gram-negative bacteria**	**64**	**5**	**31**	**4**	**33**	**6**	**20**	**10**	**15**	**8**	**18**	**3**	**26**	**4**	**37**	**5**
*Haemophilus* spp.	51	4	28	3	23	4	18	9	15	8	13	2	18	3	32	4
*Legionella* spp.	3	0	0	0	3	1	1	1	0	0	0	0	3	0	0	0
Other Gram-negative bacteria	10	1	3	0	7	1	1	1	0	0	5	1	5	1	5	1
**Anaerobic bacilli**	**1**	**0**	**0**	**0**	**1**	**0**	**0**	**0**	**0**	**0**	**1**	**0**	**0**	**0**	**1**	**0**
*Bacteroides* spp.	1	0	0	0	1	0	0	0	0	0	1	0	0	0	1	0
**Other bacteria**	**5**	**0**	**2**	**0**	**3**	**1**	**0**	**0**	**0**	**0**	**3**	**0**	**4**	**1**	**1**	**0**
**Fungi and parasites**	**143**	**10**	**81**	**10**	**62**	**11**	**14**	**7**	**13**	**7**	**77**	**11**	**73**	**11**	**69**	**10**
*Candida* spp.	99	7	64	8	35	6	13	7	10	5	51	7	49	8	49	7
*Aspergillus* spp.	33	2	11	1	22	4	0	0	3	2	21	3	18	3	15	2
Other fungi and parasites	11	1	6	1	5	1	1	1	0	0	5	1	6	1	5	1
**Viruses**	**3**	**0**	**1**	**0**	**2**	**0**	**0**	**0**	**0**	**0**	**3**	**0**	**3**	**0**	**0**	**0**

ECDC: European Centre for Disease Prevention and Control; HAP: healthcare-associated pneumonia; LRT: lower respiratory track; spp.: species.

**^a^**European Union countries, plus Croatia (EU Member State since 1 July 2013), Iceland and Norway.

^b^Excluding 302 isolates due to HAP at admission or due to missing dates.

^c^PN1–PN3: bacteriologic diagnostic test for pneumonia performed by positive quantitative culture from minimally (PN1) or possibly (PN2) contaminated LRT specimen, or by alternative microbiological methods (PN3) (see Box); PN4: positive sputum culture or non-quantitative LRT specimen culture (see Box).

The distribution of microorganisms differed significantly between IAP and non-IAP HAP cases (p < 0.001). Gram-positive cocci were isolated less frequently from IAP cases than non-IAP cases (15% vs 24%) and non-fermenting Gram-negative bacteria were isolated more frequently (40% vs 30%). The largest differences were for *Acinetobacter* spp. (13% vs 5%) and *S. aureus* (10% vs 15%) (Table 2).

Similarly, the distribution of microorganisms differed by time of onset of HAP (p < 0.001). Gram-positive cocci were isolated less frequently in HAP with late onset (27% at ≤ 4 days, 23% at 5–7 days and 16% at ≥ 8 days). Gram-negative bacteria were more frequent in HAP with late onset (30%, 36% and 39%, respectively). The largest differences between ≤ 4 vs ≥ 8 days of onset per microorganism were seen for *Acinetobacter* spp. (4% vs 11%) and *Haemophilus* spp. (9% vs 2%), and differences generally did not exceed 10% (Table 2).

There was little difference in the distribution of microorganisms from patients fulfilling the case definitions PN1–PN3 (obtained specimens less likely to be contaminated) vs those in category PN4 (obtained specimens more likely to be contaminated) (p = 0.07) (Table 2).

Antimicrobial susceptibility testing results for selected microorganism–antimicrobial combinations were available for 841 isolates, mostly showing a high proportion of non-susceptibility. For example, 91 (81%) of 112 isolates of *Acinetobacter* spp. were non-susceptible to carbapenems and 73 (47%) of 157 *S. aureus* isolates were resistant to meticillin (i.e. MRSA). Carbapenem-non-susceptibility was common among *P. aeruginosa* (81 of 204 isolates; 40%) (Table 3).

**Table 3:** Antibiotic resistance testing among selected microorganisms isolated in healthcare-associated pneumonia, ECDC point prevalence survey in acute care hospitals, European Union/European Economic Area^a^, 2011–2012 (n = 841 isolates)

Selected pathogens and antimicrobial resistance	Total		Intubation-associated pneumonia					Time of onset						
	n resistant or non-susceptible / N tested	% resistant or non-susceptible	Yes		No/Unknown		p value	≤ 4 days		5–7 days		≥ 7 days		p value
			n resistant or non-susceptible / N tested	% resistant or non-susceptible	n resistant or non-susceptible / N tested	% resistant or non-susceptible		n resistant or non-susceptible / N tested	% resistant or non-susceptible	n resistant or non-susceptible / N tested	% resistant or non-susceptible	n resistant or non-susceptible / N tested	% resistant or non-susceptible	
Antimicrobial-resistant Gram-positive cocci^b^	76 / 177	43	30 / 83	36	46 / 94	49	NS	4 / 31	13	12 / 27	44	43 / 81	53	0.007
*Staphylococcus aureus,* resistant to meticillin (MRSA)	73 / 157	47	30 / 73	41	43 / 84	51	NS	4 / 30	13	12 / 23	52	40 / 71	56	0.004
*Enterococcus* spp., resistant to vancomycin	3 /20	15	0 / 10 (0)	0	3 / 10	30	NS	0 / 1	0	0 / 4	0	3 / 10	30	NS
Antimicrobial-resistant non-fermenting Gram-negative bacteria^c^	172 / 316	54	128 / 219	58	44 / 97	45	0.04	7 / 27	26	16 / 34	47	113 / 191	59	0.01
*Pseudomonas aeruginosa*, non-susceptible to carbapenem	81 / 204	40	53 / 132	40	28 / 72	39	NS	4 / 23	17	4 / 18	22	55 / 122	45	0.04
*Acinetobacter* spp., non-susceptible to carbapenems	91 / 112	81	75 / 87	86	16 / 25	64	0.005	3 / 4	75	12 /16	75	58 / 69	84	NS
Enterobacteriaceae, non-susceptible to third-generation cephalosporins	139 / 348	40	91 /224	41	48 / 124	39	NS	16 / 57	28	9 / 44	20	81 / 175	46	0.02
Enterobacteriaceae, non-susceptible to carbapenems	38 / 336	11	25 / 214	12	13 / 122	11	NS	4 / 55	7	1 / 41	2	24 / 170	14	NS
*Klebsiella* spp., non-susceptible to carbapenems	32 / 128	25	19 / 81	23	13 / 47	28	NS	2 / 25	8	0 / 11	0	23 / 64		0.003
*Escherichia coli*, non-susceptible to carbapenems	3 / 88	3	3 / 55	5	0 / 33	0	NS	1 / 11	9	0 / 14	0	1 / 44	2	NS
Total	**387 / 841**	**46**	**249 / 526**	**47**	**138 / 315**	**44**	**NS**	**27 / 115**	**23**	**37 / 105**	**35**	**237 / 447**	**53**	**< 0.001**

ECDC: European Centre for Disease Prevention and Control; NS: not statistically significant (p > 0.05); spp.: species.

**^a^**European Union countries, plus Croatia (EU Member State since 1 July 2013), Iceland and Norway.

**^b^**Limited to meticillin-resistant *Staphylococcus aureus* or vancomycin-resistant *Enterococcus* spp.

**^c^**Limited to carbapenem-non-susceptible *Pseudomonas aeruginosa* or carbapenem-non-susceptible *Acinetobacter* spp.

There were some differences in the proportion of antimicrobial resistance / non-susceptibility between isolates from IAP and non-IAP HAP cases, as well as associations between these proportions and the time of onset of infection. Significant differences by time of onset were identified for meticillin resistance among *S. aureus*, for carbapenem-non-susceptibility among *Klebsiella* spp. and *P. aeruginosa*, and for non-susceptibility to third-generation cephalosporins among Enterobacteriaceae, as shown in Table 3. Finally, carbapenem-non-susceptibility among *Acinetobacter* spp. was more frequent among IAP than non-IAP HAP cases (Table 3).

### Antimicrobial use

Of the 2,902 patients with HAP, 2,471 (85%) were receiving 3,453 antimicrobials as therapy for HAP; one patient with HAP was receiving five different antimicrobials. An additional 379 (13%) HAP cases were receiving antimicrobials for reasons other than HAP.

Most of the antimicrobials prescribed to treat HAP were penicillins (30% of 3,453 antimicrobials) including combinations of penicillins with beta-lactamase inhibitors (24%), extended-spectrum penicillins without anti-pseudomonal activity (3%) and beta-lactamase inhibitors (2%). The second most commonly prescribed group of antimicrobials was other beta-lactam antibacterials (24%) including carbapenems (12%), third-generation cephalosporins (8%) and second-generation cephalosporins (3%). The third most commonly prescribed group of antimicrobials was the unspecific group of ‘other antibacterials’ (14%), including glycopeptide antibacterials (6%), polymyxins (4%) and imidazole derivatives (3%). Other prescribed antimicrobials were fluoroquinolones (10%), aminoglycosides (5%); antimycotics for systemic use (5%), macrolides, lincosamides and streptogramins (5%), tetracyclines (2%), and sulphonamides and trimethoprim (2%). Antimicrobials for the treatment of tuberculosis, intestinal anti-infectives, combinations of antibacterials and amphenicols were reported in less than 1% of HAP cases.

To obtain an overview of empiric antimicrobial therapy choices for HAP, we performed a sub-analysis of antibiotic regimens for HAP that were given to patients without microbiologically confirmed HAP (‘PN5’) and stratified them between IAP and HAP without known intubation before symptom onset (non-IAP HAP). We also excluded those receiving antibiotic treatment for other reasons than a HAP. There were 1,203 patients included in this sub-analysis; 220 had an IAP and 983 had a non-IAP HAP. Among patients with an IAP, the most frequently prescribed single antimicrobial group was the combination of penicillins, including beta-lactamase inhibitors (24%), followed by carbapenems (9%), third-generation cephalosporins (7%) and second-generation cephalosporins (6%). An additional 35% of the patients received combinations of antimicrobial groups. Among patients with a non-IAP HAP, the most frequently prescribed single antimicrobial group was the combination of penicillins, including beta-lactamase inhibitors (35%), followed by fluoroquinolones (8%), third-generation cephalosporins (6%) and carbapenems (5%). The combination of several antimicrobial groups was prescribed for 28% of the patients.

In addition, 874 patients who did not meet the case definition for a HAP were reported as receiving antimicrobial treatment for HAP according to the treating physician. Of these, 533 (61%) were in the United Kingdom (UK) and Ireland.

## Discussion

The ECDC PPS showed that on the day of the survey, one in 80 hospitalised patients in EU/EEA countries is diagnosed with a HAP (prevalence: 1.3%; 95% CI: 1.2–1.3), with a nearly 7 times higher prevalence in ICUs (8.1%; 95% CI: 7.4–8.9) and marked differences between other specialities. As expected, patients with longer hospital stay, older patients and men had a higher prevalence of HAP.

Approximately one in six intubated patients had a HAP on the day of the ECDC PPS (15%; 95% CI: 14–17); however, two thirds of all patients with a HAP had not been intubated in the 48 hours before the onset of their HAP. Additionally, 15% of patients with a HAP who were intubated on the day of the survey were intubated after the onset of the HAP. This confirms that while intubation remains the main risk factor for HAP, HAPs also occur in non-intubated patients [25]. Surveillance of all HAPs, including non-intubated HAP cases, is required to assess the full burden of HAPs. Prevention of HAP should also take into account non-intubated patients.

The number of hospitalised patients developing a HAP during hospital stay in EU/EEA countries is high. HAPs are associated with a high case fatality and long-term adverse clinical outcomes. With about 269 disability-adjusted life years (DALYs) per 100,000 population, they represent the largest fraction of the overall burden of disease of HAIs and they contribute a higher burden of disease for the EU/EEA than any of the other 32 communicable diseases under surveillance at EU/EEA level and studied in [1,26]. ECDC estimates that there are 702,315 (95 %CI: 664,764–744,419) cases of HAP each year in EU/EEA countries, resulting in an estimated 26,972 (CI: 21,859–32,541) attributable deaths [1].

The HAP prevalence estimated here for the EU/EEA is generally within the prevalences estimated in other studies. Most recent PPSs from Europe and North America reported a prevalence of HAP or healthcare-associated lower respiratory tract infection of around 1% [5-12,17] and up to 2.8% [13-17], which is generally consistent with data from the ECDC PPS; however, the HAP prevalence was slightly lower (0.9%) in a large PPS in the US [9]. This difference is unlikely to be explained by different case definitions, as they have been shown to be comparable [27], but may be linked to longer hospital stays in the ECDC PPS (median time between admission and survey date: 6 days) [18] than the US PPS (median time between admission and survey date: 3 days), which would increase the risk of developing and chances of detecting a HAP.

A relatively high number (n = 874) of patients, many of them in the UK and Ireland, suffered a HAP according to the clinicians’ judgement, but did not fulfil the ECDC PPS case definition. This could either indicate a problem with the implementation of the rather complex HAP case definition used in the ECDC PPS, or it may simply be due to differences in the surveillance and clinical case definitions, as they have different objectives. Clinical case definitions are likely to be more sensitive, in order to avoid undertreatment. Further studies would be needed to determine the true cause for this difference in the number of HAP cases.

Microbiological results were present for only 40% of the cases, which is much lower than in the US PPS (87 / 110 (79%) HAP) [9], which may indicate differences in diagnostic practices or study procedures. However, the three most common microorganisms (*Staphylococcus aureus, Klebsiella* spp., *P. aeruginosa*) were the same in the two PPSs. Furthermore, some of the identified microorganisms are rare causes of pneumonia (e.g. coagulase negative *S. aureus*, *Enterococcus* spp. and *Candida* spp.) and isolation of these microorganisms may reflect contamination or colonisation rather than infection. Nevertheless, since a definite decision of the underlying aetiology of HAP was not possible, we decided to present these in the results section.

As expected for HAP, a large proportion of the identified microorganisms were antimicrobial-resistant. Some of the multiresistant pathogens have a high potential for further spread and tend to cause nosocomial outbreaks. By design, PPSs over-represent patients with long hospital stays, who are also at increased risk of infections with resistant microorganisms. In addition, in the ECDC PPS, antimicrobial resistance data were more frequently available for patients with longer hospital stays. Despite these possible biases, the high proportion of carbapenem-non-susceptibility (81% of *Acinetobacter* spp., 40% of *P. aeruginosa* and 11% of Enterobacteriaceae) is worrying. Common treatments, such as with a combination of penicillins, including beta-lactamase inhibitors, may be ineffective against such carbapenem-non-susceptible microorganisms, limiting treatment options and making the use of last-line antimicrobials for empirical treatment necessary.

Even though we found significant differences in the distribution of microorganisms between IAP and non-IAP HAP cases and an association between their distribution and time of onset, these differences were relatively small, only rarely exceeding 10%. In contrast, larger differences were seen in non-susceptibility for selected antimicrobials, between late- and early-onset HAP cases or between IAP and non-IAP HAP cases. However, these data should be interpreted with caution because of the PPS design, which means an over-representation of patients with longer hospital stays and an over-representation of patients with HAIs, because they require longer treatment.

In addition, the specific design of PPSs does not make them suited to identify causal pathways. As with other surveillance activities, PPSs are limited in the number of collected variables to assess risk factors. For example, co-morbidities are commonly assessed by the McCabe score, which is a highly subjective tool. A PPS is therefore limited in its usefulness to produce concrete recommendations for specific prevention strategies, but it remains an important item in the toolbox for the prevention of HAPs and other HAIs [3].

Inter-country comparisons of the prevalence of HAP from this study should be made with caution. Firstly, the ECDC PPS was not powered for the outcome of only HAP, but for all HAIs. Secondly, many countries were not able to include a representative sample of acute care hospitals. Thirdly, despite training sessions for data collectors, there may have been differences in the implementation of the ECDC PPS protocol in participating countries and acute care hospitals, which may have influenced the sensitivity and specificity of ECDC PPS results. Indeed, four countries conducted validation studies on the same day as their ECDC PPS, comparing data collection by primary data collectors to a national, external team. The country estimates for the sensitivity of HAI detection differed greatly (range: 58%–94%). Some differences in the estimated national HAP prevalences may have been due to national differences in the consequences for reporting HAIs, such as a current or historical risk to the reporter [18].

Nevertheless, the ECDC PPS provided the first ‘snapshot’ of HAIs across EU/EEA countries, confirming that HAIs are a major health threat for acute care hospitals in the region. The ECDC PPS also collected data on the structures and process indicators of infection prevention and control, thereby highlighting intervention options for local and national policymakers [18]. Additionally, a repeat of the ECDC PPS in 2017‒18 will provide an update on HAPs in Europe. In conclusion, the ECDC PPS confirms that HAP is a frequent HAI, especially among intubated patients but also commonly among non-intubated patients, requiring continued prevention efforts. Our data provide a reference as basis for future prevalence of HAPs at various settings.

## Notes

**ECDC PPS study group:** Austria: Reinhild Strauss (Federal Ministry of Health and Women, Vienna, Austria); Belgium: Karl Mertens (Sciensano, Brussels, Belgium); Bulgaria: Elina Dobreva (National Center of Infectious and Parasitic Diseases, Sofia, Bulgaria); Croatia: Ana Budimir (School of Medicine University of Zagreb, University Hospital Centre Zagreb, Zagreb, Croatia); Cyprus: Avgi Hadjiloucas (Medical and Public Health Services, Nicosia, Cyprus); Czech Republic: Jana Prattingerová (Regional Public Health Authority Liberec, Liberec, Czech Republic); Denmark: Brian Kristensen (National Center for Infection Control, Statens Serum Institut, Copenhagen, Denmark); ECDC: Dominique L. Monnet (European Centre for Disease Prevention and Control, Solna, Sweden); Estonia: Pille Märtin (West-Tallinn Central Hospital, Tallinn, Estonia); Finland: Outi Lyytikäinen, National Institute for Health and Welfare, Helsinki, Finland; France: Marine Giard (CClin Sud-Est, Lyon, France; current address: Direction de la Santé, Papeete, Polynésie française); Germany: Brar Piening (Charité – Universitätsmedizin Berlin, Berlin, Germany); Greece: Evangelos I Kritsotakis (University of Sheffield, Sheffield, UK & Cretan Infection Control Network, Heraklion, Greece); Hungary: Andrea Kurcz (Ministry of Human Capacities, Budapest, Hungary); Iceland: Ólafur Guðlaugsson (Landspítali Háskólasjúkrahús, Reykjavík, Iceland); Ireland: Karen Burns (Health Protection Surveillance Centre, Beaumont Hospital & Royal College of Surgeons in Ireland, Dublin, Ireland); Italy: Maria Luisa Moro (Regional Health and Social Agency Emilia Romagna, Bologna, Italy); Latvia: Uga Dumpis (Pauls Stradins Clinical University Hospital, Riga, Latvia); Lithuania: Jolanta Ašembergienė (Institute of Hygiene, Vilnius, Lithuania); Luxemburg: Elisabeth Heisbourg (Direction de la Santé, Luxembourg, Luxembourg); Malta: Deborah Xuereb (Mater Dei Hospital, Valletta, Malta); Netherlands: Titia Hopmans (Centre for Infectious Disease Control, Bilthoven, The Netherlands); Norway: Nina Kristine Sorknes (Norwegian Institute of Public Health, Oslo, Norway); Poland: Aleksander Deptuła (Ludwik Rydygier Collegium Medicum in Bydgoszcz, Nicolaus Copernicus University, Poland); Portugal: José-Artur Paiva (Centro Hospitalar S. Joao, University of Porto, Porto, Portugal); Romania: Roxana Serban (Institute for Public Health, Bucharest, Romania); Slovakia: Slavka Litvová (Regional Public Health Authority, Trenčín, Slovakia); Slovenia: Jana Kolman (National Institute of Public Health, Ljubljana, Slovenia); Spain: Javier Lozano (University Hospital of Burgos, Burgos, Spain); Sweden: Johan Struwe (Public Health Agency of Sweden, Solna, Sweden); UK – England: Susan Hopkins (Public Health England, London, United Kingdom); UK – N. Ireland: Lorraine Doherty (Public Health Agency, Belfast, Northern Ireland); UK – Scotland: Jacqui S Reilly (Health Protection Scotland and Glasgow Caledonian University, Glasgow, Scotland); UK – Wales: Wendy Harrison (Public Health Wales, Cardiff, Wales).
